# Task-invariant networks interfere with and task-specific networks support memory formation: An fMRI meta-analysis

**DOI:** 10.1162/IMAG.a.1119

**Published:** 2026-02-03

**Authors:** Hongkeun Kim

**Affiliations:** Department of Rehabilitation Psychology, Daegu University, Gyeongsan-si, Gyeongsangbuk-do, Republic of Korea

**Keywords:** fMRI, episodic memory, encoding, mind-wandering, intrinsic networks

## Abstract

Why do some moments imprint themselves in memory while others vanish without a trace? This meta-analysis identifies a dissociation in large-scale brain networks during encoding: networks associated with impairing encoding are task-invariant, whereas those supporting it are task-specific. Drawing on 56 functional magnetic resonance imaging (fMRI) studies employing the subsequent memory paradigm, the analysis contrasted neural activity for later-remembered versus later-forgotten trials across verbal and pictorial tasks. Using Yeo et al.’s 17-network parcellation, the results show that encoding-impairing effects consistently recruit specific subsystems within the default mode, frontoparietal, and ventral attention networks—a pattern consistent with distraction or mind-wandering. Conversely, encoding-supporting effects diverge by task: verbal encoding engages language-related networks, whereas pictorial encoding activates visuo-perceptual systems. This asymmetry suggests that encoding failure may arise from similar attentional lapses across contexts, whereas successful encoding requires precise, context-sensitive neural engagement. Taken together, these findings provide a network-level perspective on how the brain shifts between states conducive to remembering and states conducive to forgetting.

## 1 Introduction

### 1.1 Study purpose

Why do only some moments become lasting memories, while others fade away unnoticed? Despite the brain’s continuous exposure to a flood of sensory inputs, only a select subset is ultimately retained. This phenomenon—marked by moment-to-moment variability in memory encoding—is well established in experimental paradigms, where participants routinely remember only a portion of previously encountered stimuli. One possibility is that this variability reflects fluctuations in sustained attention, which dynamically modulate the allocation of cognitive resources across time (Blondé et al., 2022; deBettencourt et al., 2018; Kim, 2011, 2025; Maillet & Rajah, 2014; Shrager et al., 2008; Weidemann & Kahana, 2021). Experiences encountered during focused attention are more likely to be successfully encoded, whereas those occurring during attentional lapses are more likely to be forgotten. This suggests that the brain’s ability to form memories depends not only on mechanisms that promote encoding but also on competing systems that undermine it.

The present meta-analysis of functional magnetic resonance imaging (fMRI) studies investigates the task-specificity of encoding-supporting and encoding-impairing neural systems. Drawing on studies employing the subsequent memory paradigm—which contrasts brain activity during the encoding of subsequently remembered versus forgotten items (Wagner et al., 1998)—it seeks to clarify how large-scale networks facilitate or impair encoding. Prior work (Kim, 2025) has demonstrated that both supporting and impairing effects are distributed across multiple intrinsic networks. Building upon these findings, the current study addresses an important yet unresolved question: Do encoding-impairing networks engage consistently across different tasks, while encoding-supporting networks dynamically adapt to task-specific demands? These questions are guided by the hypothesis that encoding-impairing effects relate to attentional lapses and mind-wandering, whereas encoding-supporting effects reflect focused task engagement, as elaborated below.

### 1.2 Study design and analytical framework

Differences between tasks are defined based on the nature of the encoding stimuli—verbal versus pictorial. Although encoding tasks vary widely within each domain, those using verbal materials typically emphasize linguistic or conceptual judgments (e.g., lexical, syllable, or animacy decisions), whereas pictorial tasks more often require perceptual judgments (e.g., indoor/outdoor decisions for scenes). These tendencies likely recruit partially distinct neurocognitive processes. Moreover, even when tasks are nominally matched across domains (e.g., natural/man-made decisions), the processing of words and pictures is unlikely to rely on fully overlapping neural mechanisms. Accordingly, distinguishing verbal from pictorial domains provides a useful basis for assessing whether encoding-impairing and encoding-supporting effects reflect task-specific or task-invariant processes. The balanced representation of both domains in the available literature also supports a well-powered meta-analytic comparison.

The present analysis emphasizes network-level interpretations, consistent with the view that cognitive processes arise from coordinated activity across large-scale brain networks (Eickhoff et al., 2018; Menon & D’Esposito, 2022; Petersen & Sporns, 2015; Raichle et al., 2001). Although regional effects are also examined, the primary focus is on distributed engagement patterns evaluated using the widely adopted 17-network parcellation of Yeo et al. (2011). This framework provides a fine-grained subdivision of canonical networks, enabling precise identification of both encoding-supporting and encoding-impairing effects. By assessing both types of effects within a common network framework, the study aims to determine whether these two forms of activity show different patterns for verbal versus pictorial tasks.

Finally, the study employs meta-analysis of fMRI research as its primary methodological approach. Given the rapidly expanding body of fMRI studies using the subsequent memory paradigm, meta-analysis is well suited to identifying reliable neural patterns across diverse experimental contexts (Fox et al., 2014). This capacity to extract robust signatures from heterogeneous datasets is particularly valuable here, as subsequent memory effects show substantial variability across studies due to differences in experimental procedures and analytical approaches.

### 1.3 Research hypotheses

The present study hypothesizes a dissociation in the task-specificity of encoding-impairing versus encoding-supporting activity. If encoding-impairing effects arise from attentional lapses and mind-wandering, they would be expected to involve consistent, task-invariant networks. Mind-wandering encompasses a broad range of task-unrelated mental states, including future planning, current concerns, and external distractions (Baird et al., 2011; Seli et al., 2017; Smallwood & Schooler, 2015). The diffuse and nonspecific nature of mind-wandering suggests that similar neural mechanisms may operate across different encoding contexts. Prior meta-analytic work (Kim, 2025), which aggregated data across diverse encoding tasks, identified four networks consistently linked to encoding-impairing effects: Subnetwork A of the default mode network (DMN), Subnetworks B and C of the frontoparietal network (FPN), and Subnetwork B of the ventral attention network (VAN). Accordingly, if encoding-impairing activity reflects shared internal processes, these networks should be similarly engaged during both verbal and pictorial encoding tasks.

In contrast, if encoding-supporting activity reflects focused task engagement, it is expected to vary systematically with task demands, recruiting neural networks aligned with the specific cognitive operations required by each task. Previous meta-analytic work (Kim, 2025) identified four networks associated with encoding-supporting effects: Subnetworks B and C of the DMN, Subnetwork A of the FPN, and Subnetwork A of the dorsal attention network (DAN). Among these, DMN-B—often linked to linguistic and conceptual processing (Chiou et al., 2023; Jackson et al., 2019; Kim, 2024a; Nozari & Thompson-Schill, 2016)—is expected to show preferential engagement during verbal encoding. Conversely, DAN-A and DMN-C, which are predominantly situated in visuo-perceptual regions, should demonstrate greater involvement during pictorial encoding. Subnetwork A of the FPN, given its anatomical overlap with the multiple-demand system (Duncan, 2010), is expected to show relatively limited task-specific modulation.

**Fig. 1.**
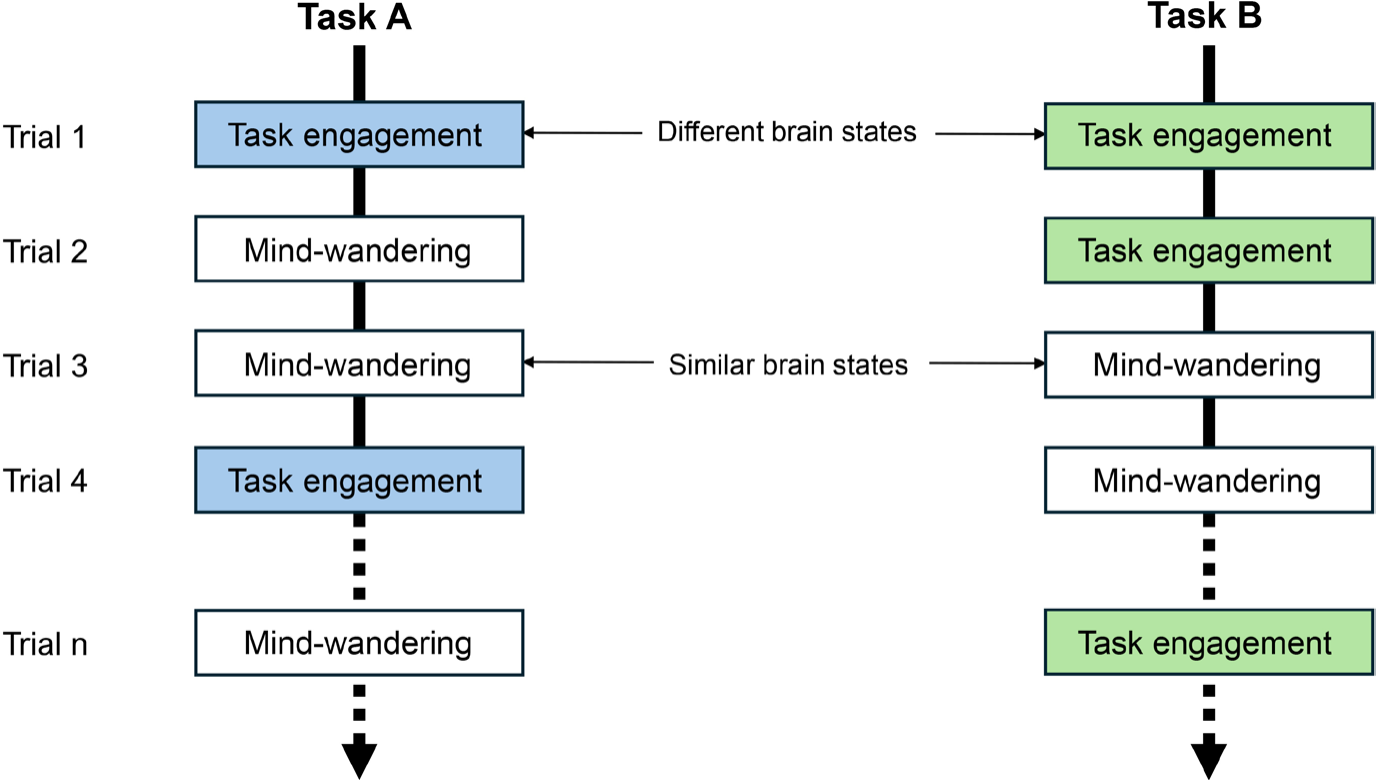
Illustration of the hypothesized task-invariant encoding-impairing effects and task-specific encoding-supporting effects. During both Task A and Task B, participants cycle between task-focused states and mind-wandering states. The task-focused states are task-specific, engaging networks tuned to the demands of each task (e.g., verbal vs. pictorial processing), consistent with the encoding-supporting hypothesis. By contrast, the mind-wandering states are task-invariant, exhibiting similar internally oriented brain activity regardless of the task from which they arise, consistent with the encoding-impairing hypothesis. This schematic illustrates how the two hypothesized effects map onto the alternation between task-focused and mind-wandering brain states.

These contrasting predictions—task-invariant networks for encoding-impairing effects and task-specific networks for encoding-supporting effects—are depicted in Figure 1. The figure highlights how states of task engagement differ across encoding types (e.g., verbal vs. pictorial), drawing on specialized neural systems tailored to distinct cognitive demands. In contrast, episodes of mind-wandering, whether occurring during verbal or pictorial encoding, are expected to consistently recruit a common set of task-invariant networks associated with stimulus-independent thought and internally oriented cognitive states. By delineating the network-level substrates underlying both encoding-facilitative and encoding-interfering effects, this study aims to clarify how the brain dynamically shifts between states leading to remembering and those leading to forgetting, reflecting fluctuations between externally focused task engagement and internally generated thought.

Finally, although two hypotheses are articulated—one for encoding-impairing effects and one for encoding-supporting effects—the primary emphasis of this study is on the former. No prior work has directly compared encoding-impairing activity across verbal and pictorial domains. By contrast, the hypothesis concerning task-specific encoding-supporting activity has been partially examined in earlier meta-analytic work (Kim, 2011, 2024b), which identified regional dissociations but did not evaluate fine-grained subnetwork-level organization or employ the stricter methodological controls used here. Thus, while the present study addresses both hypotheses, its principal contribution is to provide an initial systematic characterization of the task-specificity of encoding-impairing activity. The encoding-supporting hypothesis, although evaluated more rigorously than in prior work, is included chiefly to contextualize the encoding-impairing effects that form the primary focus of the study.

## 2 Methods

### 2.1 Study identification and selection

Eligible studies were identified using a multi-pronged search strategy. First, a systematic search of the PubMed database was conducted in April 2025 using the keywords: fMRI AND (“subsequent memory” OR “memory encoding”). A parallel search using similar terms was performed on Google Scholar. In addition, studies included in a recent meta-analysis of subsequent memory (Kim, 2025) were reviewed for potential inclusion. All studies were screened based on a set of predefined inclusion criteria, described below.

#### 2.1.1 Core criteria

To qualify for inclusion in the meta-analysis, studies were required to meet the following core criteria: (a) use of fMRI as the primary measurement modality; (b) inclusion of neurologically healthy participants only (including healthy control groups within clinical studies); (c) reporting of activation coordinates in standardized stereotactic space, either Talairach or Montreal Neurological Institute (MNI); and (d) implementation of the subsequent memory paradigm as the foundation for contrast generation.

#### 2.1.2 Stimulus modality

Only studies employing visually presented stimuli were eligible. To enable a clear distinction between verbal and pictorial encoding conditions, studies were excluded if verbal and pictorial stimuli were either presented together within a single trial (e.g., word–scene pairs) or presented in separate trials but analyzed as a single, undifferentiated condition.

#### 2.1.3 Encoding task variability

Encoding-phase tasks across studies were diverse, typically involving simple semantic or perceptual judgments (e.g., animacy judgments, indoor/outdoor decisions). No restrictions were placed on task type, ensuring broad generalizability of the findings across encoding paradigms.

#### 2.1.4 Contrast types

To capture both encoding-supporting and encoding-impairing effects, the meta-analysis included a broad range of subsequent-memory designs, including old/new recognition, memory confidence ratings, source memory, free or cued recall, and remember/know judgments. These paradigms contributed contrasts that clearly differentiated high- from low-quality encoding trials, such as remembered versus forgotten, high-confidence remembered versus forgotten, source- and item-remembered versus item-remembered only, recalled versus not recalled, and recollected versus forgotten. Crucially, only studies reporting both directions of each comparison—high quality relative to low quality and low quality relative to high quality—were retained, ensuring that encoding-supporting and encoding-impairing effects could be identified within the same experimental context.

#### 2.1.5 Whole-brain coverage

Only studies reporting whole-brain results were included. Studies restricted to predefined regions of interest (ROIs) or relying solely on small-volume correction (SVC) were excluded, as coordinate-based meta-analytic methods assume that all brain voxels carry an equal a priori probability of activation. When a study reported both whole-brain and ROI/SVC findings, the whole-brain results were used; ROI/SVC activations were additionally retained only if they satisfied the statistical thresholds applied in the whole-brain analysis.

### 2.2 Characteristics of included studies

A total of 30 verbal and 26 pictorial studies met all inclusion criteria. Brief summaries of each dataset are provided below, with full details available in the Supplementary Material.

The verbal dataset included 34 encoding-impairing contrasts (low-quality > high-quality) and 34 encoding-supporting contrasts (high-quality > low-quality), drawn from 30 independent samples comprising 672 participants. Several studies contributed more than one contrast from the same participant group, producing a larger number of contrasts than independent samples. In total, these contrasts yielded 202 encoding-impairing and 305 encoding-supporting activation foci. Stimuli consisted of single words (24 contrasts), word pairs (9), and sentences (1). Encoding tasks included animacy judgments, semantic classification, syllable counting, mental imagery, silent reading, and related variants. Memory outcomes were derived from confidence ratings (10 contrasts), old/new recognition (7), source memory tasks (6), remember/know procedures (6), and cued recall tests (5).

The pictorial dataset comprised 31 encoding-impairing contrasts and 31 encoding-supporting contrasts, drawn from 26 independent samples involving 902 participants. These contrasts contributed 300 encoding-impairing and 273 encoding-supporting activation foci. Stimulus types included scenes (15 contrasts), object images (12), faces (1), and mixed or collapsed stimulus categories (3). Encoding tasks included indoor/outdoor classification, mental imagery, animacy judgments, water-presence judgments, and related paradigms. Memory performance was assessed using confidence ratings (11 contrasts), old/new recognition (9), source memory tasks (9), free recall (1), and cued recall (1).

### 2.3 Meta-analytic procedures

All meta-analyses were conducted using the Activation Likelihood Estimation (ALE) algorithm (Eickhoff et al., 2009), as implemented in GingerALE version 3.02 (http://www.brainmap.org/ale). This coordinate-based method assesses whether activation foci from independent studies converge in specific brain regions more often than would be expected by chance. Coordinates originally reported in Talairach space were transformed into MNI space using GingerALE’s built-in conversion utility. To preserve statistical independence, contrasts derived from the same participant sample were combined into a single composite entry prior to analysis, ensuring that each participant group contributed only one effect to the model.

Separate ALE analyses were performed for encoding-supporting and encoding-impairing datasets within each task domain (verbal and pictorial), yielding four primary meta-analytic maps. Each activation focus was modeled as the center of a three-dimensional Gaussian probability distribution, with the full width at half maximum (FWHM) automatically adjusted as a function of sample size such that larger samples were assigned smaller uncertainty estimates (i.e., smaller kernels), following the ALE implementation described by Eickhoff et al. (2009). This procedure produced median FWHMs of 9.33 mm for the verbal dataset and 9.14 mm for the pictorial dataset. ALE scores were then computed at each voxel by summing the modeled activation probabilities across all reported foci. Statistical significance was determined by comparison to an analytically derived null distribution. A cluster-level familywise error correction was applied at *p* < .05, using a voxel-level cluster-forming threshold of *p* < .005. For visualization, thresholded ALE maps were projected onto an inflated cortical surface using the Population-Average, Landmark-, and Surface-based atlas (Van Essen, 2005).

Power recommendations for ALE analyses (Eickhoff et al., 2016) indicate that at least 17–20 studies are required to reliably detect moderate effects. Both the verbal (30 studies) and pictorial (26 studies) datasets exceeded this benchmark, providing sufficient statistical power for robust inferences.

### 2.4 Mapping meta-analytic effects to intrinsic networks

**Fig. 2.**
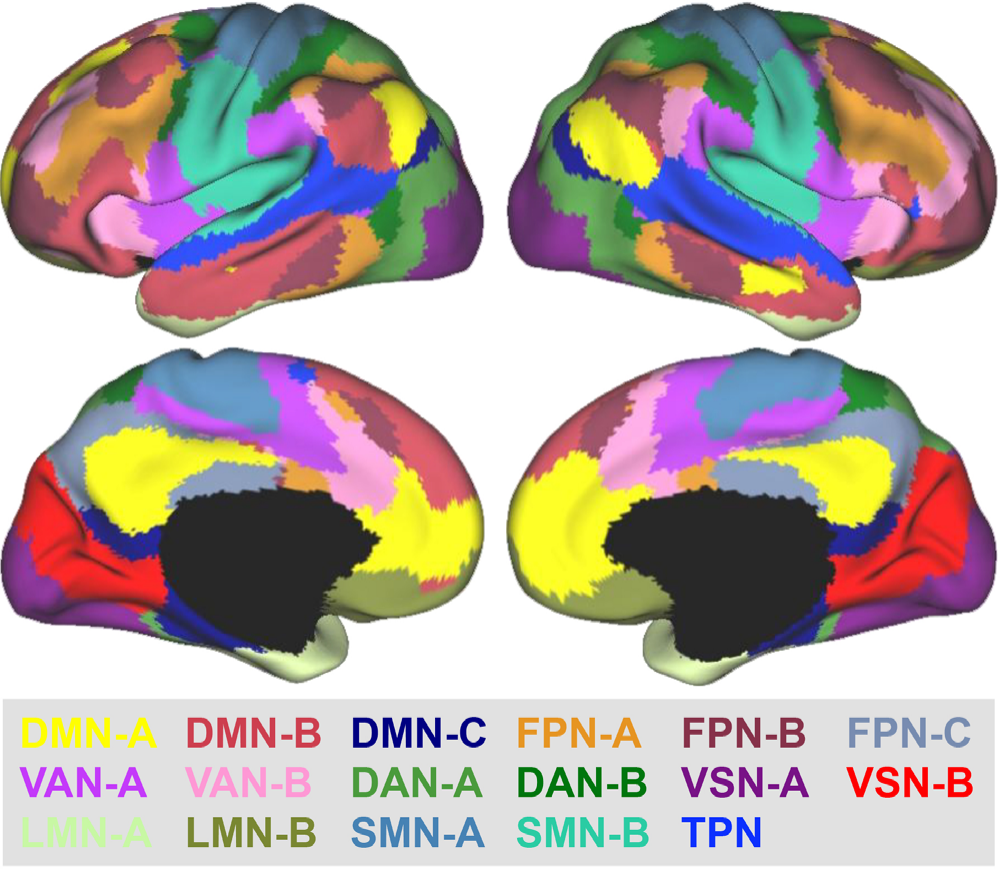
The 17-network parcellation from Yeo et al. (2011), displayed with network labels based on a slightly modified version of the naming convention employed by Kong et al. (2019). The figure has been adapted from Yeo et al. and is reprinted with permission from the American Physiological Society. DAN = Dorsal Attention Network; DMN = Default Mode Network; FPN = Frontoparietal Network; LMN = Limbic Network; SMN = Somatomotor Network; TPN = Temporal Parietal Network; VAN = Ventral Attention Network; VSN = Visual Network.

To evaluate how meta-analytic effects aligned with intrinsic brain networks, ALE maps were compared against the 17-network parcellation developed by Yeo et al. (2011). Because the original atlas did not assign formal labels to individual networks, a slightly modified version of the labeling scheme used by Kong et al. (2019) was adopted. This scheme subdivides the DMN and FPN into three subnetworks each: the VAN, DAN, visual network (VSN), limbic network (LMN), and somatomotor network (SMN) into two each; and the temporal parietal network (TPN) into one. The complete set of network subdivisions is illustrated in Figure 2.

To quantify the spatial correspondence between observed effects and intrinsic networks, the Network Association Score (Kim, 2024c) was employed. This metric is defined as:

$$Network\;Association\;Score=\left(\frac{Overlapping\;Area\;Between\;Effect\;and\;Network\text{​}}{Total\;Network\;Area}\right)\;\times 100$$

By normalizing for network size, the score allows for direct comparison across networks of differing spatial extent. Higher values reflect stronger spatial alignment between the effect and the corresponding network.

To assess the statistical significance of Network Association Scores, a nonparametric permutation procedure was used. For each effect, the number of voxels showing significant meta-analytic convergence was first identified, and these voxels were then randomly reassigned 10,000 times across the 17-network parcellation to generate an empirical null distribution reflecting no preferential network alignment. Observed Network Association Scores were evaluated against this distribution, with significance defined as *p* < .001. Unlike raw overlap counts, this approach adjusts for both network size and the overall extent of the effect, providing a more accurate and statistically principled estimate of network involvement. It also permits the detection of meaningful associations even when convergence clusters are small, provided their spatial concentration exceeds chance levels.

## 3 Results

### 3.1 Encoding-impairing networks

**Fig. 3.**
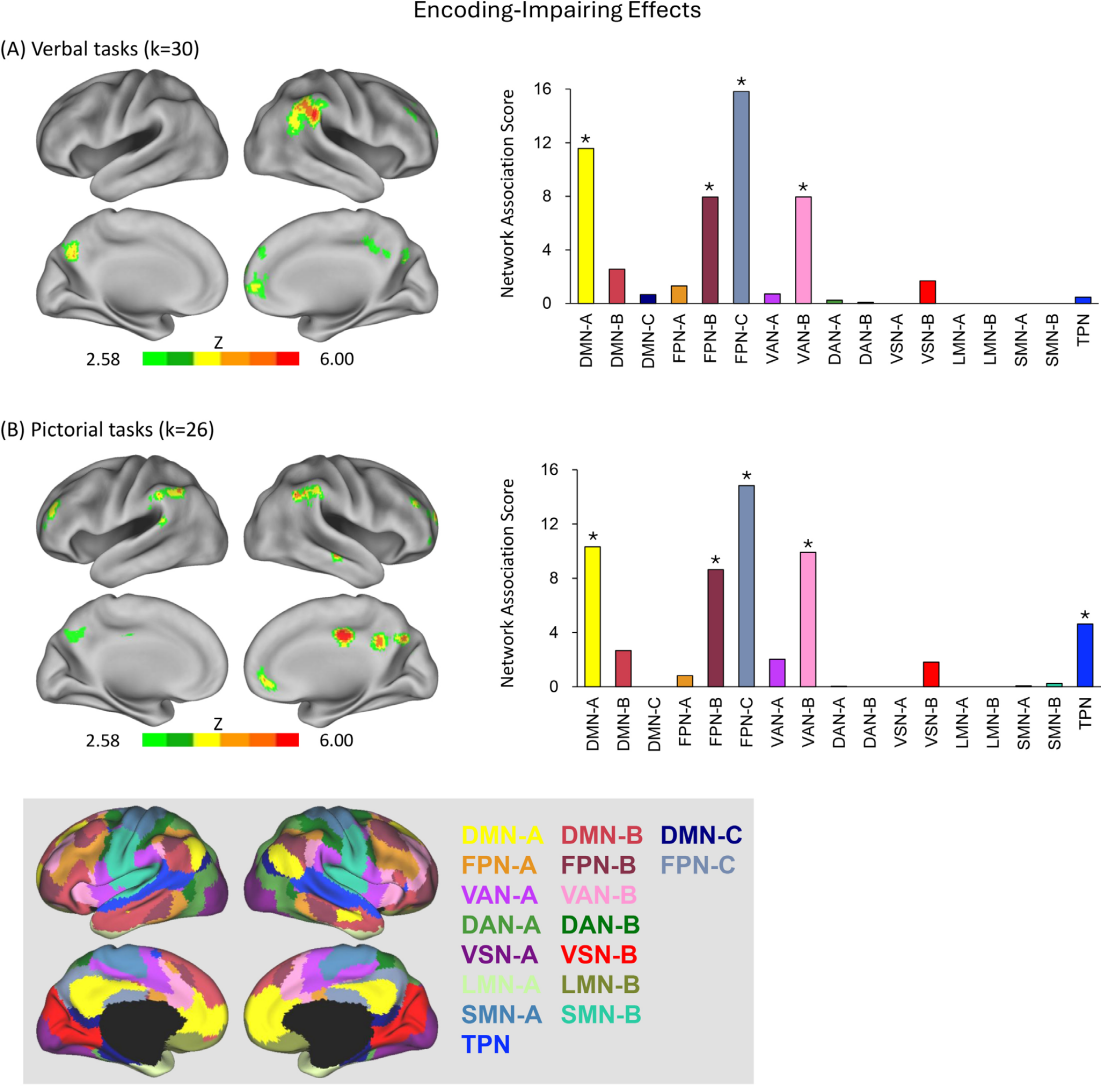
Separate analyses of encoding-impairing effects in verbal and pictorial encoding tasks. (A) Brain regions significantly associated with encoding-impairing effects during verbal tasks, along with their Network Association Scores (NAS). (B) Corresponding results for pictorial tasks. Asterisks (*) indicate statistically significant NAS values (*p* < .001). The bottom panel provides a reference map of the Yeo 17-network parcellation used for all NAS computations. *k* = number of studies included in the meta-analysis; additional abbreviations are defined in the Figure 2 legend.

ALE meta-analyses were conducted to identify regions where encoding-related activity was greater for low-quality than high-quality memory outcomes, analyzed independently for verbal and pictorial tasks. A detailed summary of the results is provided in Table 1 and visualized in Figure 3.

**Table 1.** ALE meta-analysis of encoding-impairing effects in verbal and pictorial tasks.

			Peak (MNI)				
Lobe	L/R	Region	x	y	z	Z	Volume (mm^3^)
*Verbal tasks (k = 30)*							
Frontal	R	Dorsolateral PFC	24	54	34	5.09	4,912
	R	Dorsolateral PFC	38	38	38	4.69	2,144
	R	Anteromedial PFC	6	52	6	4.48	3,272
Parietal	R	Temporoparietal junction	58	-42	34	6.69	8,168
	B	Precuneus	-8	-68	32	4.92	6,056
*Pictorial tasks (k = 26)*							
Frontal	R	Dorsolateral PFC	24	58	22	4.74	4,424
	L	Dorsolateral PFC	-36	40	24	4.50	1,808
	R	Anteromedial PFC	6	42	0	4.79	1,776
Parietal	R	Precuneus	10	-66	34	5.80	6,600
	L	Temporoparietal junction	-62	-42	38	4.93	4,048
	R	Temporoparietal junction	56	-52	40	7.59	3,496
	B	Mid-cingulate cortex/precuneus	2	-22	36	6.98	2,688
Temporal	R	Middle temporal cortex	62	-22	-10	4.96	1,856

B = bilateral; k = number of independent studies analyzed; L = left; PFC = prefrontal cortex; R = right.

For verbal encoding, significant convergence was observed primarily in the right hemisphere, with clusters centered in the right temporoparietal junction, dorsolateral and anteromedial prefrontal cortices, mid-cingulate cortex, and bilaterally in the posterior precuneus (Fig. 3A, left).

In the pictorial condition, convergence was more bilaterally distributed, with clusters in the bilateral temporoparietal junction, dorsolateral prefrontal cortex, posterior precuneus, and right-lateralized regions of the anteromedial prefrontal cortex, mid-cingulate cortex, and middle temporal cortex (Fig. 3B, left).

To assess how these regions aligned with large-scale intrinsic networks, Network Association Scores were computed. In both verbal and pictorial encoding, encoding-impairing effects showed significant overlap with DMN subnetwork A, FPN subnetworks B and C, and VAN subnetwork B (Fig. 3A–B, right). For pictorial encoding, an additional significant association emerged with the TPN. This pattern indicates a high degree of cross-task generalization: the same four networks were consistently implicated across both encoding conditions, with the TPN representing the only task-specific deviation. The relative ordering of associations—FPN-C > DMN-A > VAN-B > FPN-B—was also preserved across tasks, suggesting convergence not only in network identity but also in their relative contributions.

### 3.2 Encoding-supporting networks

**Fig. 4.**
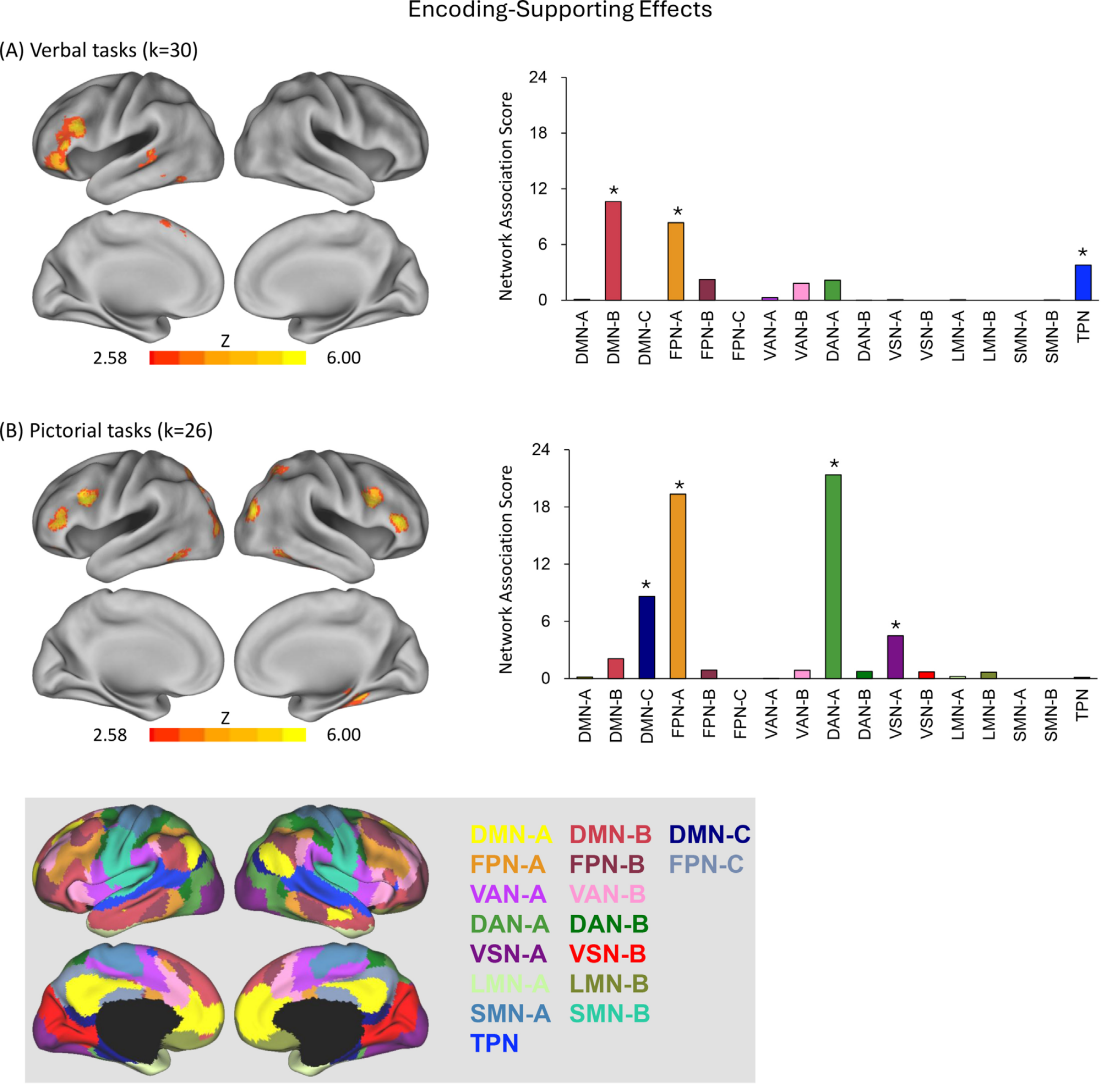
Separate analyses of encoding-supporting effects in verbal and pictorial encoding tasks. (A) Brain regions significantly associated with encoding-supporting effects during verbal tasks, along with their Network Association Scores (NAS). (B) Corresponding results for pictorial tasks. Asterisks (*) indicate statistically significant NAS values (*p* < .001). The bottom panel provides a reference map of the Yeo 17-network parcellation used for all NAS computations. k = number of studies included in the meta-analysis; additional abbreviations are defined in the Figure 2 legend.

ALE meta-analyses were also performed to identify regions where encoding-related activity was greater for high-quality than low-quality memory outcomes, again analyzed separately for verbal and pictorial tasks. Table 2 and Figure 4 summarize the results.

**Table 2.** ALE meta-analysis of encoding-supporting effects in verbal and pictorial tasks.

			Peak (MNI)				
Lobe	L/R	Region	x	y	z	Z	Volume (mm^3^)
*Verbal tasks (k = 30)*							
Frontal	L	Ventrolateral PFC	-52	30	12	5.87	15,960
	L	Superior medial PFC	-4	10	62	4.31	2,184
Temporal	L	Fusiform cortex/middle temporal cortex	-52	-48	-12	4.00	2,968
*Pictorial tasks (k = 26)*							
Frontal	L	Ventrolateral PFC	-48	32	12	5.51	3,744
	L	Inferior frontal junction	-42	8	28	6.70	3,056
	R	Inferior frontal junction	42	8	28	6.35	2,976
	R	Ventrolateral PFC	48	32	14	6.47	2,920
	L	Ventrolateral PFC	-34	36	-12	6.50	2,008
Parietal	L	Superior parietal cortex/middle occipital cortex	-24	-64	48	4.81	5,608
	R	Superior parietal cortex/middle occipital cortex	42	-80	20	6.53	5,416
Temporal	R	Parahippocampal cortex/hippocampus	30	-40	-10	4.64	3,880
	L	Fusiform cortex	-48	-56	-16	5.31	2,032
	R	Fusiform cortex	48	-56	-10	4.33	1,816

k = number of independent studies analyzed; L = left; PFC = prefrontal cortex; R = right.

In the verbal encoding condition, significant convergence was observed exclusively in the left hemisphere, with clusters localized to the left ventrolateral prefrontal cortex, superior medial prefrontal cortex, middle temporal cortex, and fusiform cortex (Fig. 4A, left).

For pictorial encoding, convergence was largely bilateral, encompassing the bilateral ventrolateral prefrontal cortex, inferior frontal junction, superior parietal cortex, fusiform cortex, and middle occipital cortex. In contrast, convergence in the parahippocampal cortex and hippocampus was right-lateralized (Fig. 4B, left).

Network Association Scores revealed distinct profiles for the two task domains. In the verbal condition, encoding-supporting regions showed significant overlap with DMN Subnetwork B, FPN Subnetwork A, and the TPN (Fig. 4A, right). In contrast, pictorial encoding recruited a largely different set of networks, including DMN Subnetwork C, DAN Subnetwork A, VSN Subnetwork A, and FPN Subnetwork A (Fig. 4B, right). Although FPN-A was engaged in both conditions, the broader network configurations diverged substantially: verbal encoding uniquely involved DMN-B and the TPN, whereas pictorial encoding selectively engaged DMN-C, DAN-A, and VSN-A. This dissociation underscores the task-specific organization of encoding-supporting networks, with only FPN-A playing a domain-general role.

## 4 Discussion

### 4.1 Task-invariant networks impair memory formation

The present findings suggest that encoding-impairing effects are linked to a stable set of neural networks—DMN-A, FPN-B, FPN-C, and VAN-B—that generalize across both verbal and pictorial encoding. Only one additional network, the TPN, showed task-specific involvement, contributing selectively during pictorial encoding. This pattern provides meta-analytic evidence for cross-task convergence in the neural correlates of encoding-impairing effects.

Such task invariance aligns with the conceptual framework in Figure 1, which attributes encoding failures primarily to internally generated cognitive states, such as mind-wandering, that arise largely independently of the specific encoding context. In contrast, accounts proposing that forgetting stems from ineffective encoding strategies (e.g., Kirchhoff & Buckner, 2006)—such as verbal recoding of nonverbal stimuli—would predict more task-specific interference. The convergence observed here, therefore, raises questions about purely strategy-based explanations and is more consistent with the view that task disengagement—rather than strategy misuse—drives encoding-impairing activity.

Several lines of prior research help clarify the roles these networks may play in impaired encoding (see Kim, 2025, for related discussion). DMN-A, centered along the midline frontal and parietal cortices, is commonly characterized as the core subsystem of the DMN. Heightened activity within this subsystem has been linked to shifts toward internally oriented thought at the expense of external stimulus processing (Andrews-Hanna et al., 2010; Buckner & Carroll, 2007; Christoff et al., 2009; Fox et al., 2015; Menon, 2023). Its involvement in encoding-impairing effects fits well with accounts proposing that memory formation weakens when attentional lapses permit internally generated thought to intrude.

FPN-B encompasses anterior and dorsolateral prefrontal regions. Its association with impaired encoding aligns with the executive-engagement hypothesis of mind-wandering (Smallwood & Schooler, 2006), which holds that sustaining internally directed thought in the presence of external task demands draws on executive control resources. Supporting this account, prior work has demonstrated coactivation of DMN and FPN regions during mind-wandering episodes occurring alongside ongoing task performance (Christoff et al., 2009; Fox et al., 2015; Smallwood et al., 2012).

FPN-C is a smaller network centered on the precuneus and mid-cingulate cortex and overlaps with the parietal memory network, whose activity has been linked to familiarity strength (Gilmore et al., 2015). From the familiarity-based perspective, FPN-C engagement during impaired encoding may reflect a familiarity signal that dampens active encoding. An alternative view, proposed by Kim (2018), argues that the network mediates the coordination of typically antagonistic internal and external cognitive states. Under this control-based account, FPN-C activation may index the cognitive demands of reconciling internally generated thought with concurrent cue processing. Further empirical work is needed to distinguish between these interpretations.

VAN-B is anchored by the temporoparietal junction and cingulo-opercular regions. Kim (2025) proposed a reduced-filtering account of its involvement in encoding-impairing effects, drawing on evidence that suppression of the VAN supports focused attention by filtering out irrelevant stimuli (Anticevic et al., 2010; Shulman et al., 2003, 2007; Solís-Vivanco et al., 2021). When VAN-B is engaged, attentional resources may be more easily captured by irrelevant internal or external inputs, fostering diffuse cognitive states that are detrimental to encoding.

Finally, the TPN was linked to encoding-impairing effects during pictorial encoding only. Localized primarily within the superior temporal cortex—a region associated with auditory processing—its involvement may reflect heightened sensitivity to environmental auditory distractions, such as scanner noise. However, the task-specific nature of this effect remains difficult to interpret and warrants further investigation.

To summarize, these interpretations offer a useful starting point for understanding how these networks contribute to encoding-impairing effects and, more broadly, to mind-wandering. However, they are preliminary and require validation in future studies.

### 4.2 Task-specific networks support memory formation

In contrast to the task-invariant nature of encoding-impairing effects, encoding-supporting activity showed clear task-dependent engagement patterns: DMN-B and the TPN were uniquely associated with verbal encoding, whereas DMN-C, DAN-A, and VSN-A were selectively engaged during pictorial encoding. Only FPN-A supported encoding across both domains. These patterns provide meta-analytic evidence that successful memory formation depends on context-sensitive neural recruitment.

For verbal encoding, DMN-B—which includes the left ventrolateral prefrontal and lateral temporal cortices—overlaps with regions consistently implicated in language and semantic processing (Badre & Wagner, 2007; Chiou et al., 2023; Jackson et al., 2019; Nozari & Thompson-Schill, 2016). Prior meta-analytic work (Kim, 2022) similarly reported preferential engagement of left inferior frontal regions during deep relative to shallow word processing. Within this framework, DMN-B involvement may reflect semantic or conceptual elaboration of verbal material (Kim, 2024a). The TPN, which includes the left superior temporal cortex, may additionally support verbal encoding through verbal rehearsal or internal auditory recoding of written input—processes thought to contribute to reading comprehension (Bemis & Pylkkänen, 2013; Henson et al., 2000).

For pictorial encoding, DMN-C—which encompasses the retrosplenial and parahippocampal cortices—is frequently linked to scene construction and spatial processing (DiNicola et al., 2020, 2023; Epstein & Baker, 2019; Epstein & Kanwisher, 1998; Mitchell et al., 2018). DAN-A, located near the parietal–occipital and temporal–occipital junctions, has been associated with top-down visual attention (Corbetta & Shulman, 2002; Sestieri et al., 2012), whereas VSN-A, located in the occipital cortex, supports perceptual analysis of visual input. Engagement of these networks is consistent with the demands of forming durable visual memories and underscores the central role of visuo-perceptual systems in successful pictorial encoding.

FPN-A, in contrast, supported memory formation across both verbal and pictorial tasks. Its spatial layout closely corresponds to the multiple-demand system described by Duncan (2010), a network reliably recruited by a wide range of cognitively demanding tasks (Assem et al., 2020; Camilleri et al., 2018; Fedorenko et al., 2013; Shashidhara et al., 2019). This cross-task involvement likely reflects domain-general executive control processes that facilitate effective encoding regardless of stimulus modality. From a complementary perspective, the role of FPN-A is also consistent with the executive failure hypothesis of mind-wandering (McVay & Kane, 2009, 2010), which posits that executive control mechanisms suppress task-unrelated thoughts and distractions. Accordingly, FPN-A recruitment may serve a dual function by sustaining controlled, goal-directed engagement with the primary encoding task while concurrently reducing interference from task-unrelated cognition.

Taken together, the encoding-supporting and encoding-impairing patterns points to functional subdivisions within both the DMN and FPN. Within the DMN, Subnetwork A was consistently linked to impairing effects, whereas Subnetworks B and C supported successful encoding in a task-dependent manner. A parallel division emerged within the FPN: Subnetworks B and C were associated with impairing effects, whereas Subnetwork A facilitated encoding across tasks. As noted earlier, this FPN division integrates both executive-engagement and executive-failure accounts of mind-wandering by linking them to distinct subnetworks. These distinctions underscore the importance of moving beyond broad network labels to consider functional specialization at the subnetwork level.

Finally, the finding that encoding-supporting networks are task-specific has implications for interpreting the apparent task invariance of encoding-impairing networks. Because ALE is optimized to detect convergent activation across studies, it may be relatively insensitive to subtle task-dependent variations. Thus, the similarity of encoding-impairing effects across verbal and pictorial tasks may partly reflect ALE’s emphasis on convergence. However, the clear task-specific patterns observed for encoding-supporting activity indicate that methodological convergence alone is unlikely to fully account for the present results. Nonetheless, it is important to acknowledge that the analytic framework used here is likely more effective at detecting robust cross-task commonalities than at capturing finer-grained, domain-specific differences.

### 4.3 Limitations and conclusions

This study has two main limitations. First, the observed network-level effects arose from specific subregions within each identified network rather than uniformly across the entire system. Although these subregions were reliably engaged, the roles of other regions within the same networks remain unclear. Given the correlated activity among network components during both tasks and rest (Cole et al., 2014; Smith et al., 2009), additional regions may contribute variably to encoding—even if not readily captured by standard subtraction-based analyses. The scope and significance of such contributions require further investigation.

Second, whereas all verbal studies involved word-based encoding, the pictorial studies encompassed diverse stimulus categories (e.g., scenes, objects, faces). Collapsing these into a single pictorial domain may obscure category-specific engagement—for example, activation of the parahippocampal place area for scenes or the fusiform face area for faces (Epstein et al., 1999; Kanwisher et al., 1997)—each associated with distinct networks. Owing to limited data, such category-level analyses were not feasible here, but future work with larger and more balanced samples may help clarify their contributions to encoding-supporting activity.

Despite these limitations, the present results support a dissociation between large-scale networks involved in memory encoding. Networks associated with encoding-impairing effects were largely task-invariant, whereas those supporting encoding showed clear task specificity. This asymmetry is notable because both effects were derived from identical task conditions and differed only in contrast direction. A plausible interpretation is that task-invariant interference reflects attentional lapses or task-unrelated cognition, whereas task-specific facilitation reflects the selective recruitment of systems tuned to the demands of the task.

This dissociation resembles classic findings from early DMN research (Shulman, Corbetta, et al., 1997; Shulman, Fiez, et al., 1997), where task > rest contrasts yielded task-specific activations, but rest > task contrasts consistently highlighted a common set of regions—later identified as the DMN (Raichle et al., 2001). The prominent involvement of both DMN and FPN regions here—distinct from early resting-state studies—may reflect the executive control required to sustain internally directed thought during ongoing tasks. Together, these findings contribute to understanding of the large-scale neural dynamics that govern shifts between mind-wandering and task-focused states and their impact on memory encoding.

## Supplementary Material

Supplementary Material

## Data Availability

The data supporting the study findings are openly available at https://identifiers.org/neurovault.collection:20692. The code used to reproduce the results is available at https://doi.org/10.13140/RG.2.2.22654.91205.

## Utilization of Generative AI and AI-Assisted Technologies in the Writing Process

While preparing this manuscript, the author used ChatGPT to improve language and readability. After using this tool, the author reviewed and edited the content as needed and takes full responsibility for the content of the publication.
