# Supplementary material for: Task-invariant networks interfere with and task-specific networks support memory formation: An fMRI meta-analysis
Source: Imaging Neurosci (Camb). 2026 Feb 3;4:IMAG.a.1119. doi: 10.1162/IMAG.a.1119 (PMC12869321; doi:10.1162/IMAG.a.1119)
Supplement: Supplementary Material [file IMAG.a.1119_supp.pdf]

Supporting information for

Task-invariant networks interfere with and task-specific networks support memory formation: An fMRI meta-analysis

Hongkeun Kim
Department of Rehabilitation Psychology, Daegu University

Table S1. Verbal encoding studies included in the meta-analysis described in the main text

Table S2. Pictorial encoding studies included in the meta-analysis described in the main text

Supplementary References. List of studies included in the meta-analyses presented in the main text

Table S1. Verbal encoding studies included in the meta-analysis described in the main text

Article	N	Stimulus per trial	Task per trial (Condition)	Subsequent memory outcomes per trial	
				Classified into high-quality encoding	Classified into low-quality encoding
Axmacher et al. (2008)	30	A word<br><br>A word	A WM task (Correct WM trials)<br>A WM task (Incorrect WM trials)	A studied word was recognized<br><br>A studied word was recognized	A studied word was not recognized<br>A studied word was not recognized
Caplan & Madan (2016)	20	A word-word pair	Memorizing	Given one word from a pair, the other word was recalled	Given one word from a pair, the other word was not recalled
Chen et al. (2013)	16	A word	Animacy judgment	A studied word was recognized	A studied word was not recognized
Clark & Wagner (2003)	17	A word	Syllable judgment	A studied word was recognized with high confidence	A studied word was not recognized
de Chastelaine & Rugg (2014)	18	A word-word pair	Relatedness judgment	An intact pair was recognized as intact	An intact pair was recognized as recombined
Duverne et al. (2009)	48	A word	Animacy judgment	A studied word was recognized with high confidence	A studied word was either unrecognized or recognized with low confidence
Fellner et al. (2016)	23	A word	Using a spatial or nonspatial mnemonic	A studied word was recalled using a mnemonic strategy	A studied word was not recalled using a mnemonic strategy
Gilead et al. (2014)	13	A sentence	Silent reading	Some thoughts linked to a sentence were recalled	No thoughts linked to a sentence were recalled
Gilmore et al. (2018)	25	A word-word pair	Memorizing	Given one word from a pair, the other word was recalled	Given one word from a pair, the other word was not recalled
Gold et al. (2006)	15	A word	Imagining an indoor or outdoor scene	Both a studied word and its task were remembered	A studied word was remembered without its task recalled
Gordon et al. (2015)	66	A word	Animacy judgment	A studied word was recognized with high confidence	A studied word was not recognized

Henson et al. (1999)	9	A word	A lexical decision task	A studied word was recognized with a 'remember' response	A studied word was recognized with a 'know' response
Herting & Nagel (2013)	17 (HF)	A word-word pair	Judging whether two words fit together	Given one word from a pair, the other word was recognized with high confidence	Given one word from a pair, the other word was not recognized
	17 (LF)	A word-word pair	Judging whether two words fit together	Given one word from a pair, the other word was recognized with high confidence	Given one word from a pair, the other word was not recognized
Kim et al. (2010)	12	Four words	A category judgment task	The number of correctly recognized words (0 to 4) was analyzed linearly	
Kirwan et al. (2008)	13	A word	Animacy or size judgment	Confidence was measured on a continuous scale and analyzed linearly	
Liu & Reder (2016)	19	A word	Recalling a previously paired word	Given a word, its paired word was recalled	Given a word, its paired word was not recalled
Maillet & Rajah (2016)	36	A word	Pleasantness or natural/man-made judgment	Both a studied word and its task were remembered	A studied word was either forgotten or remembered without its task recalled
Otten (2007)	18	A word	Size judgment (word and picture cue)	A studied word was recognized with a 'remember' response	A studied word was not recognized
		A word	Size judgment (picture cue)	A studied word was recognized with a 'remember' response	A studied word was not recognized
		A word	Size judgment (word and picture cue)	A studied word was recognized with a 'know' response	A studied word was not recognized
Otten et al. (2001); Otten & Rugg (2001)[a]	15	A word	Animacy judgment	A studied word was recognized with high confidence	A studied word was either unrecognized or recognized with low confidence
Park & Rugg (2008)	20	A word-word pair	Semantic or phonological similarity judgment	An intact pair was recognized as intact	An intact pair was recognized as either recombined or new
Raye et al. (2002)	12	A word	Silent reading (Refresh condition)	A studied word was recognized quickly	A studied word was not recognized

		A word	Silent reading (Repeat condition)	A studied word was recognized quickly	A studied word was not recognized
Reggev et al. (2016)	19	A word-word pair	Judging whether two nouns are presented	A studied word was recognized	A studied word was not recognized
Rizio & Dennis (2013)	24	A word	Intentionally directed remembering or forgetting	A studied word was recognized with a 'remember' response	A studied word was not recognized
Rizio & Dennis (2014)	23	A word	Intentionally directed remembering or forgetting	A studied word was recognized with a 'remember' response	A studied word was not recognized
Schott et al. (2006)	25	A word	Syllable judgment	A word stem was completed with a studied word	A word stem was completed with a non-studied word
Shrager et al. (2008)	14	A word	Pleasantness rating	Confidence was measured on a continuous scale and analyzed linearly	
Vannest et al. (2012)	49	A word-first letter pair (e.g., salt-p*****)	Generating a second word in each pair	A generated word was recognized	A generated word was not recognized
Wagner & Davachi (2001); Wagner et al., (1998) [a]	13	A word	Abstract/concrete judgment	A studied word was recognized with high confidence	A studied word was not recognized
Yang et al. (2015)	26	A word	Abstract/concrete judgment	A studied word was recognized	A studied word was not recognized

Note. [a] Low-quality > high-quality encoding and high-quality > low-quality encoding effects were reported in separate publications. Abbreviations: HF = high-fit participants; LF = low-fit participants; WM = working memory.

Table S2. Pictorial encoding studies included in the meta-analysis described in the main text

Article	N	Stimulus per trial	Task per trial (Condition)	Subsequent memory outcomes per trial	
				Classified into high-quality encoding	Classified into low-quality encoding
Binder et al. (2012)	59	An IAPS picture	Rating experienced arousal level	A studied picture was recalled	A studied picture was not recalled
Boenniger et al. (2021)	59	A face or scene	Memorizing	A studied item was recognized	A studied item was not recognized
Cansino et al. (2015)	12 (Y)	An object	Natural/man-made judgment	Both a studied object and its position were remembered	A studied object, but not its position, was remembered
	12 (O)	An object	Natural/man-made judgment	Both a studied object and its position were remembered	A studied object, but not its position, was remembered
Chiu & Egner (2015)	21	A face	Gender judgment	A studied face was recognized	A studied face was not recognized
Dunne & Opitz (2020)	25	Four objects	A category judgment task	A studied object was recognized	A studied object was not recognized
Elman et al. (2013)	19	A scene	Judging whether a scene includes people	A studied scene was recognized	A studied scene was not recognized
Ezzyat & Davachi (2014)	18	An object-scene and face-scene pair	Imagining an interacting scenario	Given an object or face, its paired scene was recognized with high confidence	Given an object or face, its paired scene was not recognized
Geng et al. (2022)	44	An object-character pair	Memorizing	A studied object was recognized	A studied object was not recognized
Güler & Thomas (2013)	30	An object-object pair	Memorizing	Given one object from a pair, the other object was recalled	Given one object from a pair, the other object was not recalled
Kukolja et al. (2016)	40	An object	Memorizing	A studied object was recognized	A studied object was not recognized
Kukolja et al. (2010)	18	An object	Memorizing	A studied object was recognized	A studied object was not recognized

		An object	Memorizing	Both a studied object and its position were remembered	A studied object, but not its position, was remembered
Largo (2023)	38	An object	Memorizing	Both a studied object and its position were remembered	A studied object, but not its position, was remembered
Liu et al. (2020)	35	A scene	Memorizing	Confidence was measured on a continuous scale and analyzed linearly	
Manelis et al. (2011)	13	An object	Natural/man-made judgment	A studied object was recognized with accurate detail	A studied object was not recognized
Mormino et al. (2012)	60	A scene	Judging whether a scene contains water	A studied scene was recognized with high confidence	A studied scene was not recognized
Nolden (2021)	41	A scene	Indoor/outdoor judgment (baseline test)	A studied scene was recognized	A studied scene was not recognized
		A scene	Indoor/outdoor judgment (follow-up test)	A studied scene was recognized	A studied scene was not recognized
		An object-scene pair	Judging object-scene congruence (follow-up test)	Both a studied object and its paired scene were remembered	A studied object, but not is paired scene, was remembered
Oh & Jagust (2013)	15 (Y)	A scene	Judging whether a scene contains water	A studied scene was recognized with high confidence	A studied scene was not recognized
	23 (O)	A scene	Judging whether a scene contains water	A studied scene was recognized with high confidence	A studied scene was not recognized
Poppenk et al. (2010)	13	A novel scene	Imagining an action or intention	Both a studied scene and its task were remembered	A studied scene was either forgotten or remembered without its task recalled
		A familiar scene	Imagining an action or intention	Both a studied scene and its task were remembered	A studied scene was either forgotten or remembered without its task recalled
Pruitt et al. (2021)	109	A scene	Indoor/outdoor judgment	A studied scene was recognized with high confidence	A studied scene was not recognized
Schott et al. (2023)	117	A scene	Indoor/outdoor judgment	Confidence was measured on a continuous scale and analyzed linearly	

Turk-Browne et al. (2013)	31	A scene	Memorizing	A studied scene was confidently recognized	A studied scene was confidently unrecognized
Turk-Browne et al. (2006)	16	A scene	Indoor/outdoor judgment	A studied scene recognized with high confidence	A studied scene was not recognized
Uncapher et al. (2011)	18	An object	Real/imaginary judgment (validly cued trials)	A studied object was recognized with high confidence	A studied object was not recognized
		An object	Real/imaginary judgment (invalidly cued trials)	A studied object was recognized with high confidence	A studied object was not recognized
Weis et al. (2004)	16	A scene	Building/landscape judgment	A studied scene was confidently recognized	A studied scene was confidently unrecognized

Note. IAPS = International Affective Picture System; O = older participants; Y= younger participants.

Supplementary References. List of studies included in the meta-analyses presented in the main text

Axmacher, N., Schmitz, D. P., Weinreich, I., Elger, C. E., & Fell, J. (2008). Interaction of working memory and long-term memory in the medial temporal lobe. *Cerebral Cortex*, *18*(12), 2868-2878. https://doi.org/10.1093/cercor/bhn045

Binder, J., de Quervain, D. J. F., Friese, M., Luechinger, R., Boesiger, P., & Rasch, B. (2012). Emotion suppression reduces hippocampal activity during successful memory encoding. *NeuroImage*, *63*(1), 525-532. https://doi.org/10.1016/j.neuroimage.2012.07.007

Boenniger, M. M., Diers, K., Herholz, S. C., Shahid, M., Stöcker, T., Breteler, M. M. B., & Huijbers, W. (2021). A functional MRI paradigm for efficient mapping of memory encoding across sensory conditions. *Frontiers in Human Neuroscience*, *14*, 591721. https://doi.org/10.3389/fnhum.2020.591721

Cansino, S., Estrada-Manilla, C., Trejo-Morales, P., Pasaye-Alcaraz, E. H., Aguilar-Castañeda, E., Salgado-Lujambio, P., & Sosa-Ortiz, A. L. (2015). fMRI subsequent source memory effects in young, middle-aged and old adults. *Behavioural Brain Research*, *280*, 24-35. https://doi.org/10.1016/j.bbr.2014.11.042

Caplan, J. B., & Madan, C. R. (2016). Word-imageability enhances association-memory by increasing hippocampal engagement. *Journal of Cognitive Neuroscience*, *28*(10), 1522-1538. https://doi.org/10.1162/jocn_a_00992

Chen, T.-C., Kuo, W.-J., Chiang, M.-C., Tseng, Y.-J., & Lin, Y.-Y. (2013). Over-activation in bilateral superior temporal gyrus correlated with subsequent forgetting effect of Chinese words. *Brain and Language*, *126*(2), 203-207. https://doi.org/10.1016/j.bandl.2013.05.008

Chiu, Y.-C., & Egner, T. (2015). Inhibition-induced forgetting results from resource competition between response inhibition and memory encoding processes. *Journal of Neuroscience*, *35*(34), 11936-11945. https://doi.org/10.1523/jneurosci.0519-15.2015

Clark, D., & Wagner, A. D. (2003). Assembling and encoding word representations: fMRI subsequent memory effects implicate a role for phonological control. *Neuropsychologia*, *41*(3), 304-317. https://doi.org/10.1016/s0028-3932(02)00163-x

de Chastelaine, M., & Rugg, M. D. (2014). The relationship between task-related and subsequent memory effects. *Human Brain Mapping*, *35*(8), 3687-3700. https://doi.org/10.1002/hbm.22430

Dunne, L., & Opitz, B. (2020). Attention control processes that prioritise task execution may come at the expense of incidental memory encoding. *Brain and Cognition*, *144*, 105602. https://doi.org/10.1016/j.bandc.2020.105602

Duverne, S., Motamedinia, S., & Rugg, M. D. (2009). The relationship between aging, performance, and the neural correlates of successful memory encoding. *Cerebral Cortex*, *19*(3), 733-744. https://doi.org/10.1093/cercor/bhn122

Elman, J. A., Rosner, Z. A., Cohn-Sheehy, B. I., Cerreta, A. G., & Shimamura, A. P. (2013). Dynamic changes in parietal activation during encoding: Implications for human learning and memory. *NeuroImage*, *82*, 44-52. https://doi.org/10.1016/j.neuroimage.2013.05.113

Ezzyat, Y., & Davachi, L. (2014). Similarity breeds proximity: Pattern similarity within and across contexts is related to later mnemonic judgments of temporal proximity. *Neuron*, *81*(5), 1179-1189. https://doi.org/10.1016/j.neuron.2014.01.042

Fellner, M. C., Volberg, G., Wimber, M., Goldhacker, M., Greenlee, M. W., & Hanslmayr, S. (2016). Spatial mnemonic encoding: Theta power decreases and medial temporal lobe BOLD increases co-occur during the usage of the method of loci. *eNeuro*, *3*(6), ENEURO.0184-16.2016. https://doi.org/10.1523/eneuro.0184-16.2016

Geng, F., Xu, W., & Riggins, T. (2022). Interactions between the hippocampus and fronto-parietal regions during memory encoding in early childhood. *Hippocampus*, *32*(2), 108-120. https://doi.org/10.1002/hipo.23380

Gilead, M., Liberman, N., & Maril, A. (2014). I remember thinking…": Neural activity associated with subsequent memory for stimulus-evoked internal mentations. *Social Neuroscience*, *9*(4), 387-399. https://doi.org/10.1080/17470919.2014.902862

Gilmore, A. W., Nelson, S. M., Naaz, F., Shaffer, R. A., & McDermott, K. B. (2018). BOLD activity during correct-answer feedback in cued recall predicts subsequent retrieval performance: An fMRI investigation using a partial trial design. *Cerebral Cortex*, *28*(11), 4008-4022.

https://doi.org/10.1093/cercor/bhx264

Gold, J. J., Smith, C. N., Bayley, P. J., Shrager, Y., Brewer, J. B., Stark, C. E. L., Hopkins, R. O., & Squire, L. R. (2006). Item memory, source memory, and the medial temporal lobe: Concordant findings from fMRI and memory-impaired patients. *Proceedings of the National Academy of Sciences of the United States of America*, *103*(24), 9351-9356. https://doi.org/10.1073/pnas.0602716103

Gordon, B. A., Zacks, J. M., Blazey, T., Benzinger, T. L. S., Morris, J. C., Fagan, A. M., Holtzman, D. M., & Balota, D. A. (2015). Task-evoked fMRI changes in attention networks are associated with preclinical Alzheimer’s disease biomarkers. *Neurobiology of Aging*, *36*(5), 1771-1779. https://doi.org/10.1016/j.neurobiolaging.2015.01.019

Güler, O. E., & Thomas, K. M. (2013). Developmental differences in the neural correlates of relational encoding and recall in children: An event-related fMRI study. *Developmental Cognitive Neuroscience*, *3*, 106-116. https://doi.org/10.1016/j.dcn.2012.07.001

Henson, R. N., Rugg, M. D., Shallice, T., Josephs, O., & Dolan, R. J. (1999). Recollection and familiarity in recognition memory: An event-related functional magnetic resonance imaging study. *Journal of Neuroscience*, *19*(10), 3962-3972. https://doi.org/10.1523/jneurosci.19-10-03962.1999

Herting, M. M., & Nagel, B. J. (2013). Differences in brain activity during a verbal associative memory encoding task in high-and low-fit adolescents. *Journal of Cognitive Neuroscience*, *25*(4), 595-612. https://doi.org/10.1162/jocn_a_00344

Kim, H., Daselaar, S. M., & Cabeza, R. (2010). Overlapping brain activity between episodic memory encoding and retrieval: Roles of the task-positive and task-negative networks. *NeuroImage*, *49*(1), 1045-1054. https://doi.org/10.1016/j.neuroimage.2009.07.058

Kirwan, C. B., Wixted, J. T., & Squire, L. R. (2008). Activity in the medial temporal lobe predicts memory strength, whereas activity in the prefrontal cortex predicts recollection. *Journal of Neuroscience*, *28*(42), 10541-10548. https://doi.org/10.1523/jneurosci.3456-08.2008

Kukolja, J., Göreci, D. Y., Onur, Ö. A., Riedl, V., & Fink, G. R. (2016). Resting-state fMRI evidence for early episodic memory consolidation: Effects of age. *Neurobiology of Aging*, *45*, 197-211. https://doi.org/10.1016/j.neurobiolaging.2016.06.004

Kukolja, J., Thiel, C. M., Eggermann, T., Zerres, K., & Fink, G. R. (2010). Medial temporal lobe dysfunction during encoding and retrieval of episodic memory in non-demented APOE epsilon4 carriers. *Neuroscience*, *168*(2), 487-497. https://doi.org/10.1016/j.neuroscience.2010.03.044

Largo, G. V. (2023). Investigating individual differences in the impact of variations in sustained attention during episodic memory encoding (Unpublished master's thesis). McGill University, Montreal. https://escholarship.mcgill.ca/concern/theses/8336h735r

Liu, X. L., & Reder, L. M. (2016). fMRI exploration of pedagogical benefits of repeated testing: When more is not always better. *Brain and Behavior*, *6*(7), e00476. https://doi.org/10.1002/brb3.476

Liu, Z. X., Rosenbaum, R. S., & Ryan, J. D. (2020). Restricting visual exploration directly impedes neural activity, functional connectivity, and memory. *Cerebral Cortex Communications*, *1*(1), tgaa054. https://doi.org/10.1093/texcom/tgaa054

Maillet, D., & Rajah, M. N. (2016). Assessing the neural correlates of task-unrelated thoughts during episodic encoding and their association with subsequent memory in young and older adults. *Journal of Cognitive Neuroscience*, 28(6), 826-841. https://doi.org/10.1162/jocn_a_00935

Manelis, A., Wheeler, M. E., Paynter, C. A., Storey, L., & Reder, L. M. (2011). Opposing patterns of neural priming in same-exemplar vs. different-exemplar repetition predict subsequent memory. *NeuroImage*, *55*(2), 763-772. https://doi.org/10.1016/j.neuroimage.2010.12.034

Mormino, E. C., Brandel, M. G., Madison, C. M., Marks, S., Baker, S. L., & Jagust, W. J. (2012). Aβ deposition in aging is associated with increases in brain activation during successful memory encoding. *Cerebral Cortex*, *22*(8), 1813-1823. https://doi.org/10.1093/cercor/bhr255

Nolden, S., Brod, G., Meyer, A.-K., Fandakova, Y., & Shing, Y. L. (2021). Neural correlates of successful memory encoding in kindergarten and early elementary school children: Longitudinal trends and effects of schooling. *Cerebral Cortex*, *31*(8), 3764-3779. https://doi.org/10.1093/cercor/bhab046

Oh, H., & Jagust, W. J. (2013). Frontotemporal network connectivity during memory encoding is increased with aging and disrupted by beta-amyloid. *Journal of Neuroscience*, *33*(47), 18425-18437. https://doi.org/10.1523/jneurosci.2775-13.2013

Otten, L. (2007). Fragments of a larger whole: Retrieval cues constrain observed neural correlates of memory encoding. *Cerebral Cortex*, *17*(9), 2030-2038. https://doi.org/10.1093/cercor/bhl111

Otten, L. J., Henson, R. N. A., & Rugg, M. D. (2001). Depth of processing effects on neural correlates of memory encoding: Relationship between findings from across- and within-task comparisons. *Brain*, *124*(2), 399-412. https://doi.org/10.1093/brain/124.2.399

Otten, L. J., & Rugg, M. D. (2001). When more means less neural activity related to unsuccessful memory encoding. *Current Biology*, *11*(19), 1528-1530. https://doi.org/10.1016/s0960-9822(01)00454-7

Park, H., & Rugg, M. D. (2008). Neural correlates of successful encoding of semantically and phonologically mediated inter-item associations. *NeuroImage*, *43*(1), 165-172. https://doi.org/10.1016/j.neuroimage.2008.06.044

Poppenk, J., McIntosh, A. R., Craik, F. I. M., & Moscovitch, M. (2010). Past experience modulates the neural mechanisms of episodic memory formation. *Journal of Neuroscience*, *30*(13), 4707-4716. https://doi.org/10.1523/jneurosci.5466-09.2010

Pruitt, P. J., Tang, L., Hayes, J. M., Ofen, N., & Damoiseaux, J. S. (2021). Age moderation of the association between negative subsequent memory effects and episodic memory performance. *Aging Brain*, *1*, 100021. https://doi.org/10.1016/j.nbas.2021.100021

Raye, C. L., Johnson, M. K., Mitchell, K. J., Reeder, J. A., & Greene, E. J. (2002). Neuroimaging a single thought: Dorsolateral PFC activity associated with refreshing just-activated information. *NeuroImage*, *15*(2), 447-453. https://doi.org/10.1006/nimg.2001.0983

Reggev, N., Bein, O., & Maril, A. (2016). Distinct neural suppression and encoding effects for conceptual novelty and familiarity. *Journal of Cognitive Neuroscience*, *28*(10), 1455-1470. https://doi.org/10.1162/jocn_a_00994

Rizio, A. A., & Dennis, N. A. (2013). The neural correlates of cognitive control: Successful remembering and intentional forgetting. *Journal of Cognitive Neuroscience*, *25*(2), 297-312. https://doi.org/10.1162/jocn_a_00310

Rizio, A. A., & Dennis, N. A. (2014). The cognitive control of memory: Age differences in the neural correlates of successful remembering and

intentional forgetting. *PLoS ONE*, *9*(1), e87010. https://doi.org/10.1371/journal.pone.0087010

Schott, B. H., Richardson-Klavehn, A., Henson, R. N. A., Becker, C., Heinze, H.-J., & Düzel, E. (2006). Neuroanatomical dissociation of encoding processes related to priming and explicit memory. *Journal of Neuroscience*, *26*(3), 792-800. https://doi.org/10.1523/jneurosci.2402-05.2006

Schott, B. H., Soch, J., Kizilirmak, J. M., Schütze, H., Assmann, A., Maass, A., Ziegler, G., Sauvage, M., & Richter, A. (2023). Inhibitory temporo-parietal effective connectivity is associated with explicit memory performance in older adults. *iScience*, *26*(10), 107765. https://doi.org/10.1016/j.isci.2023.107765

Shrager, Y., Kirwan, C. B., & Squire, L. R. (2008). Activity in both hippocampus and perirhinal cortex predicts the memory strength of subsequently remembered information. *Neuron*, *59*(4), 547-553. https://doi.org/10.1016/j.neuron.2008.07.022

Turk-Browne, N. B., Golomb, J. D., & Chun, M. M. (2013). Complementary attentional components of successful memory encoding. *NeuroImage*, *66*, 553-562. https://doi.org/10.1016/j.neuroimage.2012.10.053

Turk-Browne, N. B., Yi, D.-J., & Chun, M. M. (2006). Linking implicit and explicit memory: Common encoding factors and shared representations. *Neuron*, *49*(6), 917-927. https://doi.org/10.1016/j.neuron.2006.01.030

Uncapher, M. R., Hutchinson, J. B., & Wagner, A. D. (2011). Dissociable effects of top-down and bottom-up attention during episodic encoding. *Journal of Neuroscience*, *31*(35), 12613-12628. https://doi.org/10.1523/JNEUROSCI.0152-11.2011

Vannest, J., Eaton, K. P., Henkel, D., Siegel, M., Tsevat, R. K., Allendorfer, J. B., Schefft, B. K., Banks, C., & Szaflarski, J. P. (2012). Cortical correlates of self-generation in verbal paired associate learning. *Brain Research*, *1437*, 104-114. https://doi.org/10.1016/j.brainres.2011.12.020

Wagner, A. D., & Davachi, L. (2001). Cognitive neuroscience: Forgetting of things past. *Current Biology*, *11*(23), R964-R967. https://doi.org/10.1016/s0960-9822(01)00575-9

Wagner, A. D., Schacter, D. L., Rotte, M., Koutstaal, W., Maril, A., Dale, A. M., Rosen, B. R., & Buckner, R. L. (1998). Building memories: Remembering

and forgetting of verbal experiences as predicted by brain activity. *Science*, *281*(5380), 1188-1191. https://doi.org/10.1126/science.281.5380.1188

Weis, S., Klaver, P., Reul, J., Elger, C. E., & Fernandez, G. (2004). Temporal and cerebellar brain regions that support both declarative memory formation and retrieval. *Cerebral Cortex*, *14*(3), 256-267. https://doi.org/10.1093/cercor/bhg125

Yang, H., Cai, Y., Liu, Q., Zhao, X., Wang, Q., Chen, C., & Xue, G. (2015). Differential neural correlates underlie judgment of learning and subsequent memory performance. *Frontiers in Psychology*, *6*, 1699. https://doi.org/10.3389/fpsyg.2015.01699
